# One-Dimension Diffusion Preparation of Concentration-Gradient Fe_2_O_3_/SiO_2_ Aerogel

**DOI:** 10.3390/molecules23071502

**Published:** 2018-06-21

**Authors:** Ting Zhang, Haoran Wang, Bin Zhou, Xiujie Ji, Hongqiang Wang, Ai Du

**Affiliations:** Shanghai Key Laboratory of Special Artificial Microstructure Materials and Technology, School of Physics Science and Engineering, Tongji University, Shanghai 200092, China; 1730965@tongji.edu.cn (T.Z.); 14haoranwang@tongji.edu.cn (H.W.); zhoubin863@tongji.edu.cn (B.Z.); 1710867@tongji.edu.cn (X.J.); 1631874@tongji.edu.cn (H.W.)

**Keywords:** Fe_2_O_3_/SiO_2_ aerogel, concentration gradient, nanocomposites, 1d diffusion

## Abstract

Concentration-gradient Fe_2_O_3_/SiO_2_ aerogels were prepared by placing an MTMS (methyltrimethoxysilane)-derived SiO_2_ aerogel on an iron gauze with an HCl atmosphere via one-dimensional diffusion, ammonia-atmosphere fixing, supercritical fluid drying and thermal treatment. The energy dispersive spectra show that the Fe/Si molar ratios change gradually from 2.14% to 18.48% with a height of 40 mm. Pore-size distribution results show that the average pore size of the sample decreases from 15.8 nm to 3.1 nm after diffusion. This corresponds well with TEM results, indicating a pore-filling effect of the Fe compound. In order to precisely control the gradient, diffusion kinetics are further studied by analyzing the influence of time and position on the concentration of the wet gel. At last, it is found that the diffusion process could be fitted well with the one-dimensional model of Fick’s second law, demonstrating the feasibility of the precise design and control of the concentration gradient.

## 1. Introduction

Aerogels could be regarded not only as a special multi-functional material, but also as a new state of matter [1]. Nanoporous silica aerogel is normally derived from an organic precursor via the sol-gel process [2,3]. Aerogels have many unique features, such as a high specific surface area, low thermal conductivity, ultra-low sound velocity, and ultra-wide adjustable range of the refractive index and density [4,5,6,7,8,9,10]. A number of their physical properties, such as index of refraction, porosity and dielectric constant, correlate to their density. A material that changes in density continuously exhibits a corresponding change in these properties, which largely expands its application, such as its acoustic impedance, matching material and interstellar grains collector [11]. In 1992, Gerlach sintered the SiO_2_ aerogels at a temperature gradient, and the density gradient aerogel was obtained for the first time [12]. Some methods for preparing gradient-density aerogels have been reported, such as layer-by-layer gelation, sol-co-gelation and continuous formation technics [11]. Therefore, fabrication of gradient density aerogels has been an active area of research.

Furthermore, the method for preparing a density-gradient aerogel via the co-gelation of gradient gels can be used to produce the concentration-gradient aerogel. Composite materials are widely used in daily life and different industries [13,14]. However, concentration-gradient composite aerogels have rarely been studied. The reason may lie in the difficulty associated with preparing the co-gelled composite aerogels and accurately controlling the concentration. The composition-gradient composite aerogel was first presented by Jones et al. in 2000 [15]. A monolith of silica aerogel, doped with one-dimensional (1d) gradient-distributed tin oxide, was presented [15]. Additionally, a disc of silica aerogel that was doped radially with resorcinol-formaldehyde organic gel with a gradient has been prepared by Jones [16]. In our previous work, a co-gelation system, including tetramethoxysilane (TMOS), CuCl_2_·2H_2_O, acetonitrile and propylene oxide (PO), was adopted to prepare the concentration-gradient CuO/SiO_2_ aerogel [17].

The co-gelation method mentioned above is versatile and easily produces the aerogels with a high gradient. However, it is not easy to find metal oxide/silica co-gelation systems to prepare a concentration-gradient aerogel. For example, the homogeneous co-gelation of a Cu(II) compound and silica is difficult to achieve because the traditional catalysts may react with each other (e.g., HF and PO) or affect the reaction of the other precursor (e.g., the ammonium that could catalyze the silica precursor would lead to the precipitation of the copper precursor) [17]. Moreover, even if the suitable catalysts could be found, the gelation time of each component should be similar. Otherwise, only lamination, but not homogeneous, gel will be obtained. Thus, a simple method is needed to prepare the concentration-gradient aerogels.

**Figure 1:**
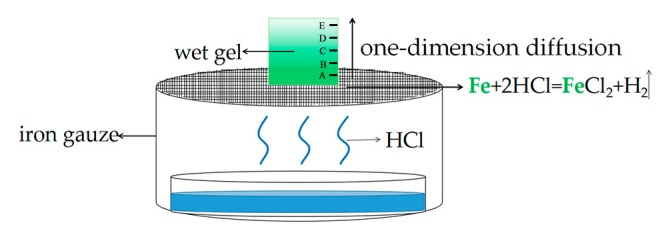
The schematic diagram of the 1d diffusion preparation of gradient Fe(II)/SiO_2_ gels.

In our previous work [5], we accidentally discovered that when the acidic MSQ gel was pin-hole dried on an iron gauze, the bottom of the wet gel turned green and the whole gel had a color gradient. We speculate that the iron gauze was corroded by acidic silica gel, causing Fe^2+^ to diffuse into the gel. In this work, we used an extra hydrochloric acid to provide an acidic atmosphere (Figure 1), so that the Fe^2+^ could diffuse into a gel of any pH value. Ammonia was used to stop the diffusion process and fix the Fe^2+^. After CO_2_ supercritical fluid drying and heat treatment, the concentration gradient Fe_2_O_3_/SiO_2_ aerogels were prepared. The above preparation method is relatively simple, and the spectrometer can be used to accurately determine the concentration information at five positions. In principle, it can be used for the diffusion of any element. In other words, one-dimensional diffusion can be used to prepare concentration-gradient aerogels with various elements. The concentration-gradient aerogels may be widely applied in the capture of hypervelocity particles, high-efficiency catalysis, high-temperature thermal insulation and many other fields [15,16].

## 2. Results and Discussion

### 2.1. Aerogel Appearance and Composition Distribution

**Figure 2:**
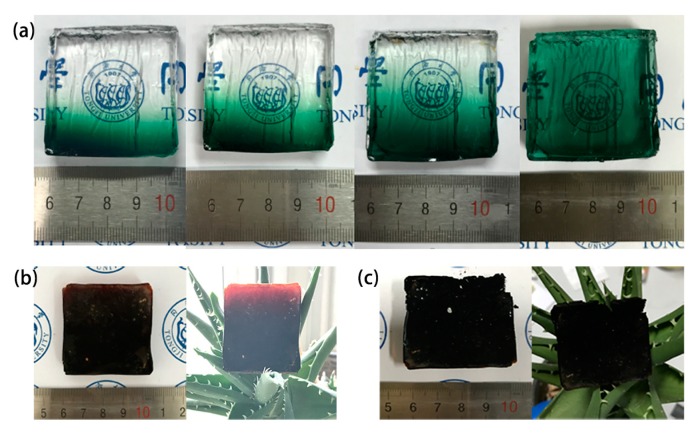
(**a**) The diffusion process of Fe^2+^ into the MSQ gel before supercritical fluid drying with the 4 × 4 × 1 cm^3^ square mold; The composites aerogel (**b**) after CO_2_ supercritical fluid drying and (**c**) after both supercritical fluid drying and thermal treatment.

The appearance of Fe_2_O_3_/SiO_2_ nanocomposites is shown in Figure 2. It can be seen that the Fe^2+^ gradually diffuse from bottom to top and finally spread to equilibrium. As shown in Figure 2a,b, the color changes from green to reddish-brown, indicating that the iron element changes from bivalent to trivalent. It is because the hydrochloric acid corrodes the iron gauze to produce Fe^2+^ that the methylsilsesquioxane (MSQ) gel becomes green. Afterwards, the ammonia reacts with Fe^2+^ to form Fe(OH)_2_, which is then oxidized into reddish-brown Fe(OH)_3_. Finally, the organic matter is burned by high-temperature calcination, and the hydroxide turns into Fe_2_O_3_ (demonstrated by the XRD patterns shown in Figure S2).

In order to further prove the composition gradient of the composite aerogel with respect to height, three points at each height were randomly tested for their EDS spectra [6]. The molar ratio of Fe/Si was calculated accordingly. The uncertainty of the molar ratio of Fe/Si in each plane was small, and the composition distribution in each plane was relatively uniform, demonstrating a good performance of the 1d diffusion process. Thus, five planes at different heights (three points in each plane) were tested to characterize the composition distribution of the composition-gradient aerogel. The molar ratio of Fe/Si at different heights is shown in Table 1. The original data are shown in Figure S3.

### 2.2. Analysis of N_2_ Adsorption-Desorption Isotherm and Pore Size Distribution

**Figure 3:**
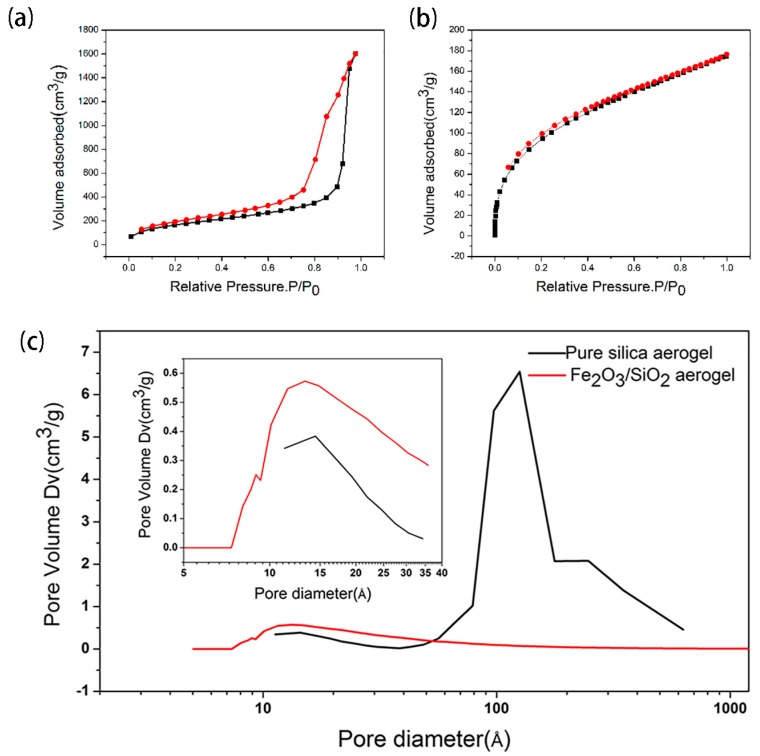
The N_2_ adsorption-desorption isotherms of: (**a**) Pure silica aerogel and (**b**) Fe_2_O_3_/SiO_2_ aerogel, and (**c**) the pore size distribution of pure silica aerogel and Fe_2_O_3_/SiO_2_ aerogel; a comparison of microporous distribution of pure silica aerogel and doped aerogel is inset.

The N_2_ adsorption-desorption isotherms and the pore size distributions of pure silica aerogel and Fe_2_O_3_/SiO_2_ aerogel (we choose the plane A to measure BET when the Fe^2+^ diffuse relatively homogeneously) are shown in Figure 3. An IV-type curve, illustrating that aerogels are typical mesoporous materials, is presented in Figure 3a. An H1-type hysteresis loop indicates that there are a large number of spherical pores stacked in succession [18,19]. An I-type curve and most microporous materials belonging to this category are shown in Figure 3b. The pore structure of the H4-type is a wedge-shaped hole in the skeleton, but more pores are concentrated in the microporous area [20]. The pore size distribution of the two samples is shown in Figure 3c. The black line represents pure silica aerogel and the red line represents Fe_2_O_3_/SiO_2_ aerogel. We can see that the pore size of silica aerogel is widely distributed across micropores, mesopores and macropores, with an average value of 15.8 nm. Moreover, the specific surface area is 526 m^2^/g. The average pore size of Fe_2_O_3_/SiO_2_ aerogel is 3.1 nm, and most pores are distributed in the region of the micropores or small mesopores. Its specific surface area is 350 m^2^/g. Obviously, the latter sample has a much smaller specific surface area and average pore size than the former. This may be because Fe-contained compounds adhere to the skeleton and fill a large amount of the pores. This decreases both the pore size and pore volume, and the specific surface area decreases accordingly. Another reason may be that the doping of iron promotes the aggregation of colloidal particles in the sol-gel system, thereby forming a relatively dense network skeleton structure. A comparison of the microporous distribution of pure silica aerogel and doped aerogel is shown in Figure 3c. It shows that the micropores of doped aerogel increase, indicating that Fe ions probably facilitate the crosslinking of the primary skeletons. The microsyneresis leads to shrinkage of the skeletons and produces more micropores [21].

### 2.3. The Microstructure of Aerogel

**Figure 4:**
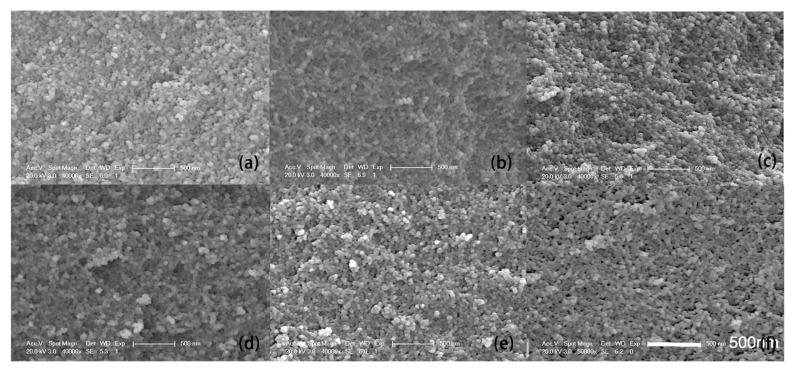
The SEM picture of the: (**a**) Pure SiO_2_ aerogel and composition-gradient Fe_2_O_3_/SiO_2_ composite aerogel at different positions: (**b**) A; (**c**) B; (**d**) C; (**e**) D; (**f**) E.

The SEM images of the SiO_2_ aerogel are shown in Figure 4a. The images of the Fe_2_O_3_/SiO_2_ aerogel, taken from different heights, represented by A, B, C, D and E, are shown in Figure 4b–f, respectively. It can be seen that there is no obvious difference in morphology between the doped aerogel and the pure aerogel. The main difference between them may be in their composition [6]. The SEM images only reflect the secondary structure of the aerogels. The secondary structure of the silica aerogels, which is difficult to change, is firmly formed before the diffusion. During the diffusion process, Fe^2+^ is dissolved in water and does not induce the pore-filling effect. However, after fixing, drying and thermal treatment processes, the Fe_2_O_3_ fills the pores, leading to an obviously higher density. For example, the density of the pure SiO_2_ aerogel and the concentration-gradient aerogel (average density) are about 140 mg/cm^3^ and 274 mg/cm^3^, respectively. The samples with high density exhibit a relatively compact stacking structure with small pores.

**Figure 5:**
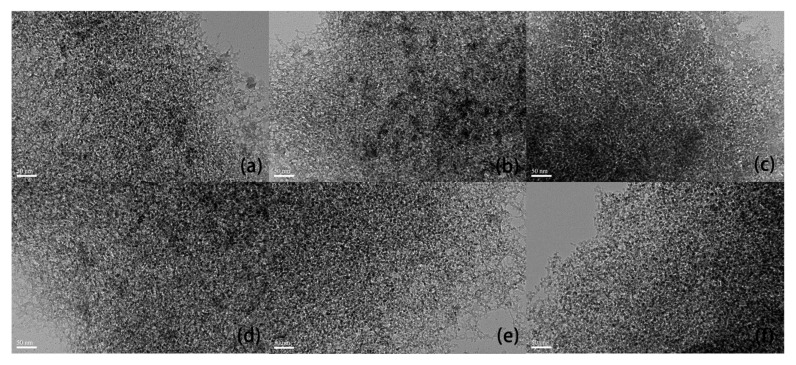
The TEM images of the: (**a**) SiO_2_ aerogel and composition-gradient Fe_2_O_3_/SiO_2_ composite. Aerogel at different positions: (**b**) A; (**c**) B; (**d**) C; (**e**) D; (**f**) E.

**Figure 6:**
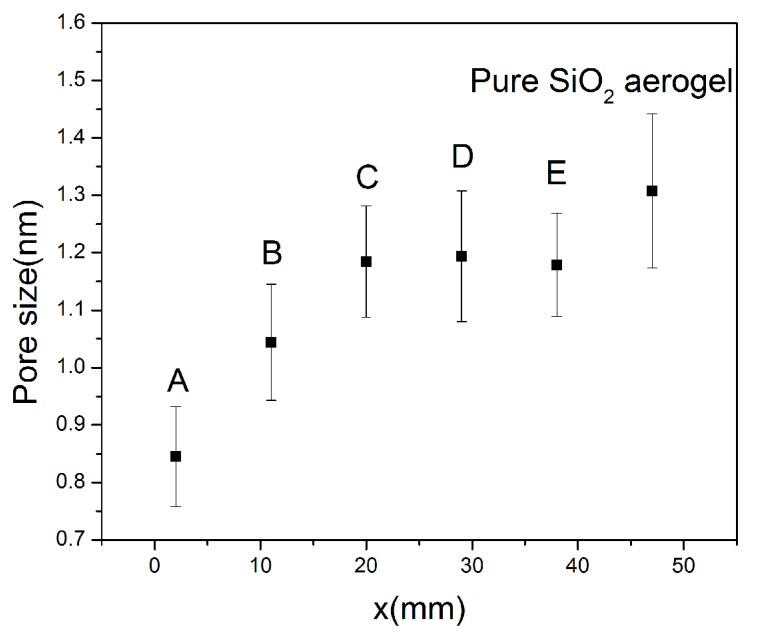
The average pore diameter of composite aerogels at five positions (A: *x* = 2 mm; B: *x* = 11 mm; C: *x* = 20 mm; D: *x* = 29 mm; E: *x* = 38 mm) and pure silica aerogel.

The TEM images of the pure SiO_2_ aerogel are shown in Figure 5a. The images of composites at positions A, B, C, D and E are shown in Figure 5b–f, respectively. As in the SEM images, it can be seen that there is not much difference in morphology between the pure SiO_2_ aerogel and the Fe_2_O_3_/SiO_2_ aerogel. The five positions are not significant different. There are many small white dots in the TEM images, indicating micropores or small mesopores, which is demonstrated in the pore-size distribution results. We randomly selected 30 pores in each TEM image, and measured their individual sizes and the average size. The pore size distributions of the sample are shown in Figure S4. As shown in Figure 6, the average pore size obviously increases gradually with the Fe compound decrease at positions A, B, and C. However, the average pore size of D and E is almost unchanged, while that of the pure silica aerogel is the largest. This is because the molar ratio of Fe/Si at positions A, B and C is 18.48%, 11.56%, 5.06%, respectively. There is a difference of ~7% between adjacent positions. The more the Fe/Si ratio is, the more pore volume is occupied, resulting in a smaller pore size. The average iron content at positions D and E are 3.61% and 2.46%, respectively. Compared with the former, the difference in iron content at the last three points is very small, which leads to the small change in the pore diameter. As a comparison, the micropore size of the pure SiO_2_ aerogel is obviously larger than that of the last three points, which is consistent with the analysis above.

### 2.4. One-Dimensional Diffusion Model of Fick's Second Law

Fick’s laws of diffusion were derived by Adolf Fick in 1855. They can be used to solve the diffusion coefficient *D* [22]. Fick’s first law applies to steady-state diffusion, while Fick’s second law applies to unsteady states. Non-steady state diffusion means that the concentration of the diffusion component changes not only with distance, but also with time. Our research is consistent with Fick’s second law. If we know the concentration distribution at any time, we can precisely design the concentration-gradient aerogels by fixing the ion at the appointed time.

**Figure 7:**
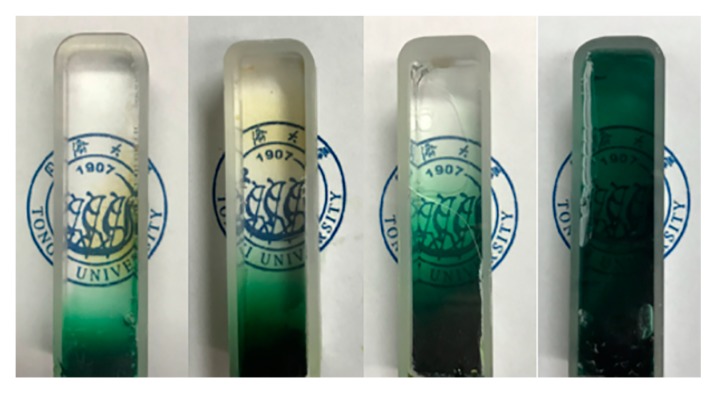
The diffusion process of Fe^2+^ into the gel before supercritical drying with the mold of cuvette.

The samples for studying the 1d non-steady state diffusion are shown in Figure 7. Since the width of the cuvette is only 1 cm, which is much smaller than that of the iron gauze, we think that there is only vertical diffusion but no horizontal diffusion in this process. That is, a one-dimensional diffusion model that conforms to Fick’s second law. The one-dimensional diffusion formula is given as Equation (1):

(1) $$\frac{\partial c}{\partial t}=D\frac{\partial^{2}c}{\partial x^{2}}$$

where *c* is the concentration, *t* is the time, *D* is the diffusion coefficient and *x* is the distance measured from the bottom of the gel. We simplified the experiment to the following model: The model is a one-dimensional infinite-length diffusion model [23,24]. It is assumed that Fe^2+^ starts to diffuse from *x* = 0 and can diffuse to infinity *x* = ∞. A simple case of diffusion is with time *t* in one dimension from a boundary located at position *x* = 0, and the concentration is maintained at a value *n*_0_. Thus, the concentration at any position and any time could be described as:

(2) $$n(x,t)=n_{0}erfc(\frac{x}{2\sqrt{Dt}})$$

where erfc is the complementary error function, *n*_0_ is the constant concentration in the position *x* = 0, *n*(*x*, *t*) is the concentration at the position *x* and *t* is the time. As a quick approximation of the error function, the first two terms of the Taylor series can be described as Equation (3):

(3) $$n(x,t)=n_{0}\left[1-2(\frac{x}{2\sqrt{D\pi t}})\right]$$

**Figure 8:**
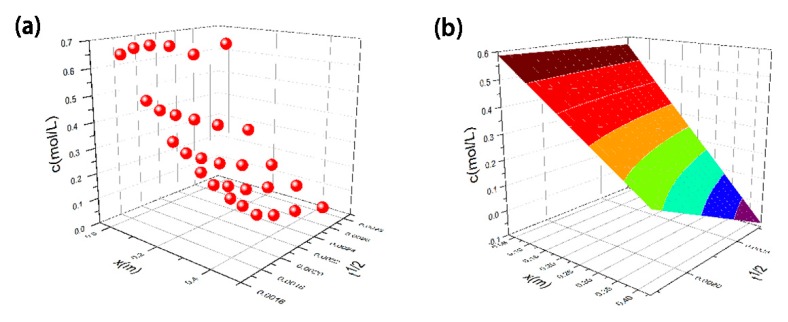
(**a**) The original experimental data and (**b**) the fitted surface according to Equation (3).

The diffusion coefficient *D* was calculated by the unsteady-state method using a nonlinear curve fitting algorithm. The first two sets of data at 12 h and 24 h are removed because the Fe^2+^ diffusion to position A and the concentrations of Fe^2+^ at positions B, C, D, and E are all 0. The original experimental data and the fitted surface according to Equation (3) are shown in Figure 8a,b, respectively. The fitting results, including *R*^2^, *n*_0_ and *D*, are listed in Table 2 (the method of calculating the concentration is shown in the support information and the calibration curve of the molar absorption coefficient *k* is shown in Figure S1). The correlation coefficient *R*^2^ is 0.89447 and the value of *D* is 3.52 × 10^−7^ m^2^/s. There are inevitable errors in the experiment. For example, the gel shrinks slightly; at the beginning, Fe^2+^ is not at every position even if the first two groups of data are removed; and this model is not exhaustive. The temperature may influence the diffusion coefficient *D*, and the higher the temperature, the faster the diffusion. Thus, we conducted these experiments with a constant temperature and humidity environment in order to avoid the influence of temperature. The concentration of HCl may only affect the boundary concentration *n*_0_. However, neither the temperature nor the HCl concentration significantly affects the microstructure of the aerogels. We find that the correlation coefficient is relatively high when considering these factors. It can be approximated that the diffusion process of Fe^2+^ into the gel conforms to the one-dimensional model of Fick’s second law.

In fact, this method could be used to precisely design any kind of oxide/silica gradient aerogels if the diffusion efficiency is measured. Thus, we think that this method is versatile and provides a new route to various metal oxide/silica concentration-gradient aerogels with designable distributions.

## 3. Experiments and Characterizations

### 3.1. Materials

Methyltrimethoxysilane (≥98%, MTMS), methanol (≥99.5%, MeOH), isopropanol (≥99.5%, IPA), hexadecyltrimethylammonium chloride (≥98%, CTAC), urea (≥99.0%), hexamethyl disiloxane (≥98%), acetic acid (≥99.5%, C_2_H_4_O_2_), ammonia hydroxide (25~28 wt%, NH_4_OH), hydrochloric acid (34.46 wt%, HCl) were all purchased from Sinopharm Chemical Reagent Co., Ltd, Shanghai, China.

### 3.2. Preparation of Silica Gels

The MTMS, deionized water, acetic acid and ammonia hydroxide were used as the precursor, solvent and catalyst, respectively. The diluted acetic acid solution (5 mM/L), CTAC, urea and MTMS were mixed at a volume ratio of 1:0.04:0.3:0.5. After 30 min of stirring and hydrolysis, the sol was poured into a 4 × 4 × 1 cm^3^ square mold. To easily demold it, a layer of dimethyl silicone oil was coated on the inner surface of the square mold, and then a layer of plastic wrap was spread over the silicone oil. Because the sol should be placed in a 60 °C drying oven to gel, thermal expansion of the gel results in a mold release difficulty. The entire diffusion process was carried out under constant temperature and humidity conditions.

### 3.3. One Dimensional Diffusion Preparation of Concentration-Gradient Gel

After being aged in a 60 °C oven for 2–3 days, the gel was demolded and placed into the simple device we designed (Figure 1). The role of the hydrochloric acid is to produce HCl vapor, which erodes the iron gauze to generate Fe^2+^. Deionized water is placed in the container in order to provide the saturated vapor and prevent the gel from cracking, since the surface evaporation of the solvent (water) leads to the collapse of the gel skeleton. During the diffusion process, the gradient of the concentration of Fe^2+^ in the gel changes from bottom to top, so we chose five different positions (from bottom to top, the positions are A, B, C, D and E, as shown in Figure 2) for characterization. We measured the absorption spectra at each position every 12 h (we selected three points at every height to reduce the error). In order to observe the diffusion processes, we poured the sol into a 4.5 × 1 × 1 cm^3^ cuvette, then gelled and finally placed it into the device shown in Figure 1.

### 3.4. Preparation of Concentration-Gradient Fe_2_O_3_/SiO_2_ Aerogel

We replaced the hydrochloric acid with diluted ammonia when Fe^2+^ diffused to the position E. The purpose is to fix Fe^2+^ and prevent the loss of the iron element during solvent replacement and CO_2_ supercritical fluid drying. After the Fe^2+^ was fixed in the gel, the solvent methanol, isopropyl alcohol and ethanol were replaced three times successively, followed by CO_2_ supercritical fluid drying. The dried sample was calcined in a muffle furnace at 600 °C for 2 h. Finally, we obtained the concentration-gradient Fe_2_O_3_/SiO_2_ aerogel.

### 3.5. Characterizations

The absorption spectrum at five positions were measured by an ultraviolet-visible-infrared (UV-Vis-IR) spectrophotometer (JASCO V-570, JASCO, Kyoto, Japan). In order to prove the gradient of the concentration of iron in the sample, an energy dispersive spectrometer (EDS, Philips-XL30FEG, Thermo Fisher Scientific Inc., Hillsboro, OR, USA) was used to measure the molar ratio of Fe/Si at different heights. The microstructure and morphology of the aerogels with different heights was characterized by a scanning electron microscope (SEM, Philips-XL30FEG, Thermo Fisher Scientific Inc., Hillsboro, OR, USA) and transmission electron microscopy (TEM, */JEM-2100F, JEOL, Tokyo, Japan). The Brunner−Emmet−Teller (BET, AUTOSORB-1-MP, Quantachrome, Boynton Beach, FL, USA) was used to measure the pore size distribution and specific surface area of the silica aerogel and the composite aerogel.

## 4. Conclusions

In this work, we have found a novel method to synthesize concentration-gradient Fe_2_O_3_/SiO_2_ nanocomposites via one-dimension diffusion. The EDS results show that the Fe/Si molar ratios change gradually from 2.14% to 18.48% with a height of 40 mm. The larger the Fe/Si molar ratio, the smaller the pore size in composites. The average pore size of the sample decreases from 15.8 nm to 3.1 nm after diffusion, indicating a pore filling effect. The SEM and TEM images show that, except for the stacking density and the micropore size, there is not much difference in microscopic appearance from five heights. At last, it is found that the diffusion process could be fitted well with the one-dimensional model of Fick’s second law. This diffusion fabrication is a simple and useful technique for preparing gradient materials. 

## Figures and Tables

**Table 1:** Molar ratio of Fe/Si at different heights of the composition-gradient aerogel.

Measure Plane	Height from the Bottom (mm)	Molar Ratio of Fe/Si (%)
Position A	2	18.48 ± 0.26
Position B	11	11.56 ± 0.29
Position C	20	5.06 ± 0.76
Position D	29	3.61 ± 0.09
Position E	38	2.14 ± 0.32

**Table 2:** The one-dimensional model of Fick’s second law.

Models	D (m^2^/s)	*n*_0_ (mol/L)	R^2^
one-dimensional model	3.52 × 10^−7^	0.61612	0.89447

## Supplementary Materials

The following are available online. Figure S1: The calibration curve of Fe^2+^ aqueous solution, Figure S2: The XRD figure of the calcined solute, Figure S3: The EDS of the five planes interval at about 9 mm (three points in each plane were randomly tested: (a) *x* = 2 mm; (b) *x* = 11 mm; (c) *x* = 20 mm; (d) *x* = 29 mm; (e) *x* = 38 mm), Figure S4: Pore size distribution of composites at different heights: (a) A; (b) B; (c) C; (d) D; (e) E and the pore size distribution of (f) pure silica aerogel.
