# Supplementary material for: One-Dimension Diffusion Preparation of Concentration-Gradient Fe2O3/SiO2 Aerogel
Source: Molecules. 2018 Jun 21;23(7):1502. doi: 10.3390/molecules23071502 (PMC6100486; doi:10.3390/molecules23071502)
Supplement: Supplementary file 1 [file molecules-23-01502-s001.pdf]

molecules

Supporting information for:

One-dimension diffusion preparation of concentration-gradient Fe_2O_3/SiO_2 aerogel

Ting Zhang [1], Haoran Wang[1], Bin Zhou[1], Xiujie Ji[1] and Hongqiang Wang[1], Ai Du [1,*]

[1] Shanghai Key Laboratory of Special Artificial Microstructure Materials and Technology, School of Physics Science and Engineering, Tongji University, Shanghai 200092, China.

* Correspondence: duai@tongji.edu.cn ; Tel.: +86 21 6598 6071

1. Measurement of molar absorption coefficient k

According to Beer's law $A=kbc$, the concentration of Fe^{2+} at different heights must have three known quantities: absorbance A, molar absorption coefficient k and absorber layer thickness *b*. The spectrophotometer was used to measure the absorbance A and b=1cm, so the molar absorption coefficient *k* needs to be calculated. The following method is used to calculate *k*: dip a completely dark green gel into a small amount of deionized water and the gel turns from the original dark green to transparent and the remaining solution is dark green (Fe^{2+} aqueous solution) after three days at room temperature. 3ml dark green solution was placed in a cuvette and its absorption spectrum A_0 was measured with a spectrophotometer (set its concentration to C_0). Continue to dilute the solution and measure its absorbance A_1, A_2...A_6. The following method is used to calculate C_0: take 43ml Fe^{2+} aqueous solution drying, calcining, grinding and then calculated C_0 = 0.232mol/L. The values were linearly fitted to give $A=0.0106+4.82513c$, R^2=0.99428(Figure S1). So the molar absorption coefficient k = 4.82513. The calcined solute was measured for EDS and XRD. The EDS results show that the average molar ratio of Si/Fe is 3.4% (select five points randomly) and in addition to these it also contains oxygen. From this we infer that the main phase of this is Fe_2O_3. Figure 2 further proves that the calcined solute is Fe_2O_3. The peak is offset slightly and some peaks are not marked because of the influence of complex silicates, but it can be shown that the main phase is Fe_2O_3.

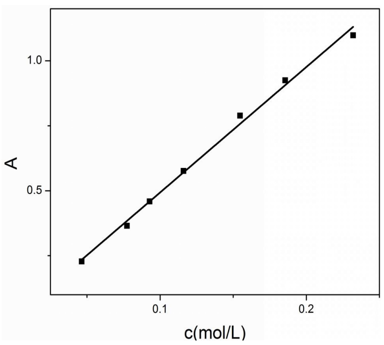

Figure S1. The calibration curve of Fe^{2+} aqueous solution.

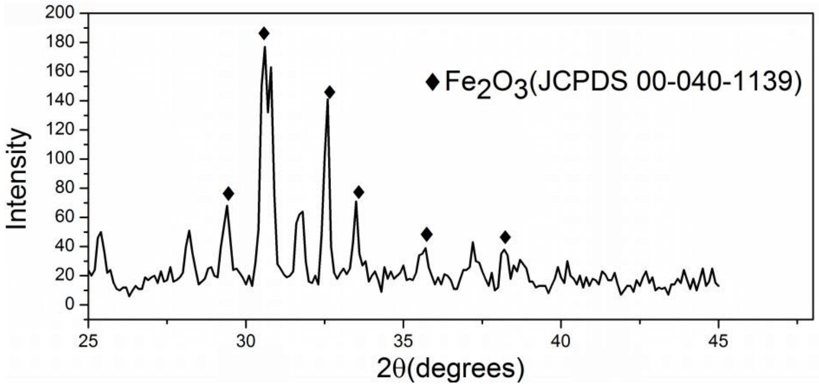

Figure S2. The XRD figure of the calcined solute.

2. Aerogel composition distribution

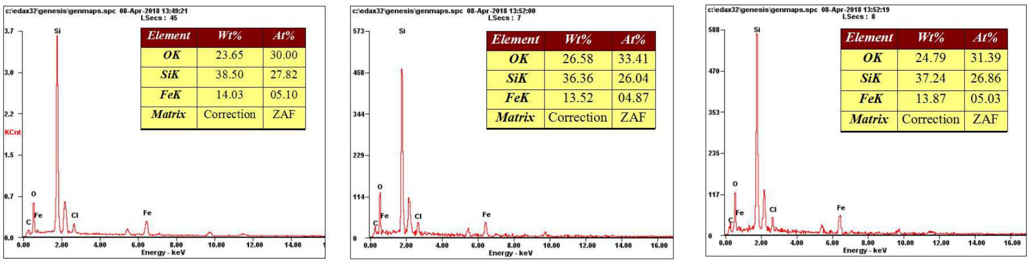

(**a**)

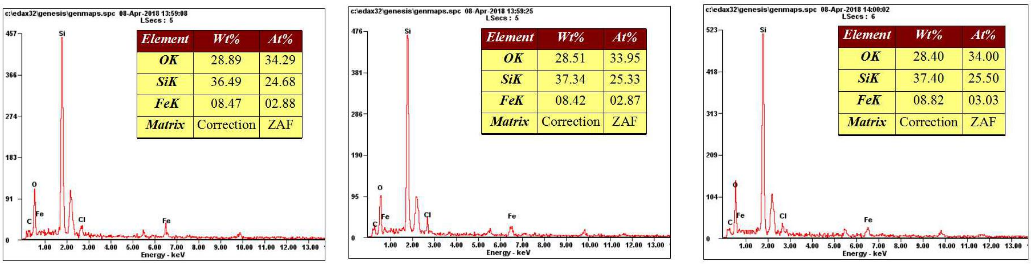

(**b**)

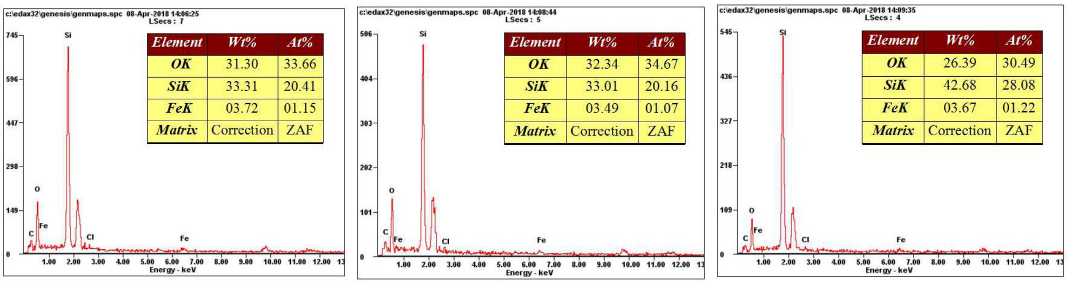

(**c**)

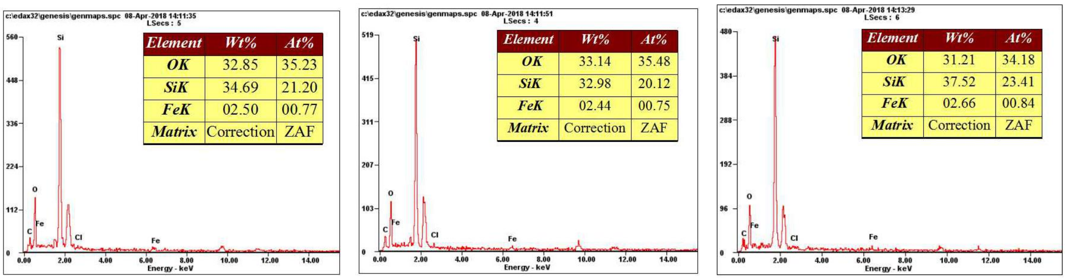

(**d**)

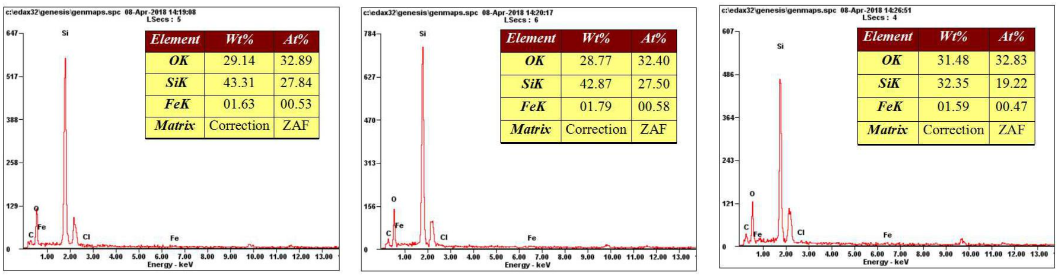

(**e**)

Figure S3. The EDS of five planes interval about 9mm, three points in each plane were taken randomly to test((**a**): *x*=2mm; (**b**): *x*=11mm; (**c**): *x*=20mm; (**d**): *x*=29mm; (**e**): *x*=38mm)

3. The average pore diameter of five heights

The following method is used to measure the average pore size of composites: magnify the TEM image of the corresponding height, then use the ImageJ software to measure the size of 30 holes which selected randomly. This software can only measure but not be marked so we don't have dimensioned figures. But we recorded all the data and made it into the following histogram as figure S4.

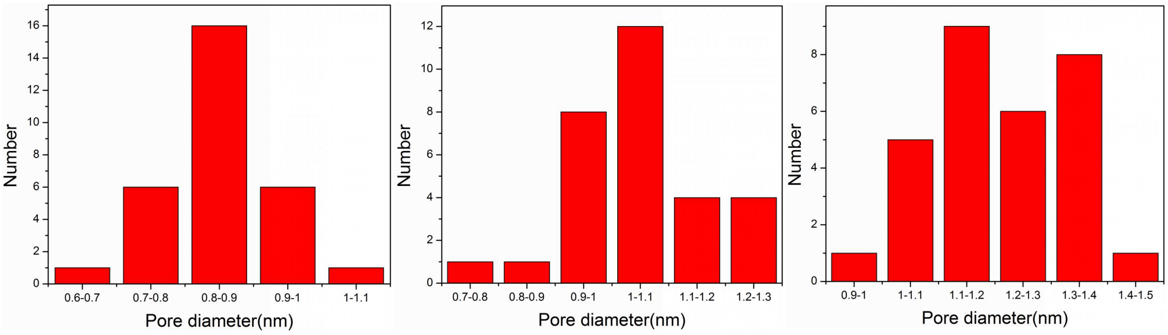

(**a**) (**b**) (**c**)

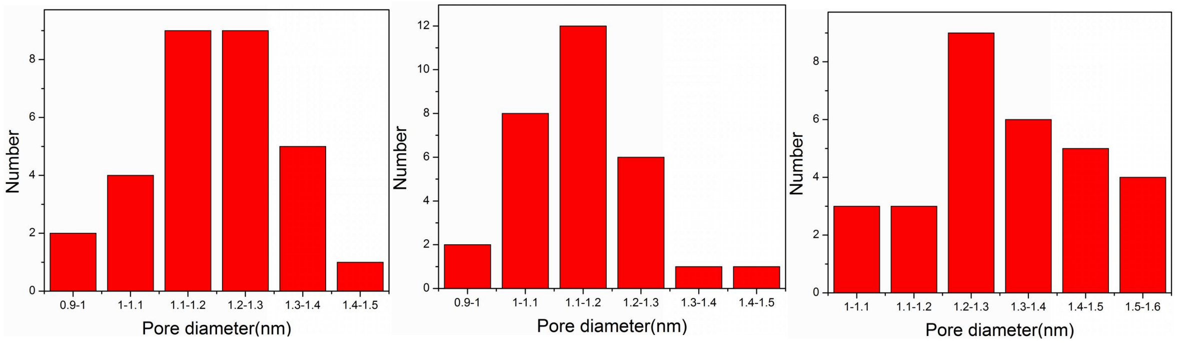

(**d**) (**e**) (**f**)

Figure S4. Pore size distribution of composites at different heights: (**a**) A; (**b**) B; (**c**) C; (**d**) D; (**e**) E and pore size distribution of (**f**) pure silica aerogel.
